# Progress in Surgery

**Published:** 1896-12-26

**Authors:** 


### Progress in Surgery.

### SURGERY OF THE INTESTINES.

**Perforating Ulcer.**—Anderson[1] records the situation of the ulcer found in twenty-one post-mortem examinations of cases which had died of rupture of the bowel following on the ulceration of typhoid fever. In eight cases it was within a foot of the ileo-cæcal valve; in six cases, between one and two feet away from the valve; in three cases, between two and three feet; in two cases, about four feet; and in two no record is made. No perforation was found in any other part of the intestinal canal. He therefore thinks that for operative purposes an incision in the right iliac region as for appendicitis is preferable to one in the mid-line of the abdomen. He considers it desirable to cover the line of sutured intestine with an omental graft. Rapidity of operation is essential. Where there is difficulty in finding the perforation it is better to cleanse the peritoneum thoroughly, and insert a gauze drain, than to depress the patient by a prolonged search for the ruptured bowel. Smith[2] records an interesting case of fatal hæmorrhage from a stercoral ulcer following an operation for the relief of intestinal obstruction from carcinoma of the ascending colon. During the necessary manipulation at the time of operation (enterostomy) a perforation of the gut was noticed in the centre of a large stercoral ulcer three inches above the obstruction. Hæmorrhage took place from this on the eleventh day after operation, and the patient rapidly sank. Warren[3] reports a case of perforating ulcer of the duodenum in a man sixty-two years of age. The symptoms were supposed, at the time of operation, to be due to perforation of the vermiform appendix. The ulcer of the duodenum was, however, discovered and closed with Lembert's suture. Death occurred on the third day, no action of the bowels having taken place after operation.

**Intestinal Obstruction.**—Adenst[4] discusses the subject of post-operative intestinal occlusion. It is generally caused by intestinal adhesions to surfaces denuded during an operation, but may be due to bands, faulty positions of intestine, intestinal spasm, and operative errors. Much importance is attached to an abnormal retiring angle at the splenic flexure, which may produce compression of the small intestine between its sides. He condemns the use of purgatives and opium, and claims that laparotomy should be performed in cases in which vomiting has followed on complete retention of flatus and fæces lasting from three to five days. The cæcum should be first examined, and, if it be distended, the sigmoid flexure should then be examined. The cause should be sought by examination of pedicles, raw surface, possible kinks of intestine, and drainage tubes. Distension of the transverse colon suggests obstruction at the splenic flexure. When the obstruction is seated at an infected centre, as after removal of suppurating uterine appendages, laparotomy should be avoided in order to prevent infection of the whole peritoneal cavity. An artificial anus should be established—(1) when the patient is *in extremis*; (2) after failure to find the cause of the obstruction; (3) if the symptoms of obstruction persist after the performance of laparotomy. Lund[5] mentions a successful case of abdominal section for obstruction due to a gall-stone in a woman aged forty-four years. Page[6] reports a successful case of operation caused by a band in a child five years of age; Murray[7] one of intestinal obstruction caused by an adherent vermiform appendix; and Butler-Smythe[8] a case of obstruction subsequent to an attack of peritonitis in which the following forms of obstruction were met and dealt with consecutively: (1) Intestine adherent to the abdominal wall; (2) volvulus; (3) omental band constricting the transverse colon; (4) peritoneal band constricting the descending colon; and (5) impaction of fæcal matter. Other similar cases are referred to by Nicoll[9].

**Intussusception** is considered by Rydygier,[10] who has been able to add eighty-four new cases to the sixty-six collected by Braun. Operative interference is as important in intussusception as in strangulated hernia. Rydygier always makes two attempts at reduction by the injection of water, and this failing, proceeds to operation. The formation of an artificial anus is only to be recommended in case of threatening collapse, for only two cases have recovered after such treatment. Entero-anastomosis is also to be excluded, for it leaves the disease unattacked. Disinvagination gives the best results, but, this failing, the Jessett-Barker method of resection is probably the best. The advantages of this method of resection are: (1) A small amount of intestine only is sacrificed; (2) the tying off of the mesentery is accomplished in one stroke; (3) the suture is applied in the shortest possible time. In cases of acute intussusception the author arrives at the following conclusions: (1) Operation must be done as soon as possible after the failure of careful bloodless methods; (2) after laparotomy, disinvagination is the method of election; (3) where disinvagination cannot be accomplished, resection is to be performed; (4) the entire mass is to be resected when the invaginating sheath shows marked changes, or where perforation is feared; (5) the formation of an artificial anus and entero-anastomosis are only justifiable when there is imminent danger of collapse. Chronic intussusception should be treated on similar lines, and temporising without good reason is bad practice, as acute changes may develop. Successful cases of laparotomy for intussusception are also reported by Lund[11] and Carpenter[12]—the latter in an infant two months old.

**Artificial Anus and Entero-Vaginal Fistula** are principally treated by resection and entero-anastomosis. Of these Chaput[13] prefers the latter, because resection is likely to lead to infection of the operative field, takes longer time, and involves the use of a circular suture, which is much inferior to entero-anastomosis in respect of solidity. The best method of preventing the fæcal matter from continuing to pass on towards the artificial anus is by ligaturing the intestine with iodoform gauze. When a fistula is discharging mucous only on the skin, it is better to close it by cauterisation with nitric acid twice a week. Harrison Cripps[14] closes a fistula by introducing within the gut a disc of indiarubber threaded with the silk. The two ends of the silk are drawn out of the fistula and tied over a small roll of lint. Brown[15] operated in one case by the following method: An incision was made round the fistula at a distance of one inch, and the skin was dissected inwards towards the opening. This flap was then stitched tightly over the opening, so as to completely shut off the intestinal aperture. Resection of the gut was then performed in the ordinary way after drawing out the intestine from the abdomen. The object of the prelimary closure by skin flaps was to prevent fæcal contamination of the peritoneum, and so rob the subsequent enterectomy of one of its chief dangers.

**Intestinal Anastomosis** is, says Bidwell,[16] required chiefly for: (1) Gangrenous bowel in strangulated hernia; (2) laceration of gut while separating extensive adhesions; (3) removal of new growths of the intestinal wall; and, (4) in the cure of an artificial anus. The general points to be considered in selecting a method of intestinal anastomosis are: (1) That adequately broad and sufficiently wide surfaces of healthy intestine should be in contact; (2) that, although it is advisable to exclude the mucous membrane from the stitches, the fibres of the submucous coat must always be included; (3) that the operation be performed as rapidly as possible; (4) that when possible the operation should be performed outside the abdominal cavity. He does not think it quite safe to entrust a patient's life to the efficiency of a spring, as in Murphy's button, though he acknowledges that this is the quickest method of operating. In favour of a bone bobbin, such as Mayo Robson's, are the following: (1) It enables the surgeon to use a continuous instead of interrupted sutures; (2) it acts as a splint to the newly sutured gut, and keeps the parts at rest; (3) it maintains a free channel through the anastomosis. The method by Paul's tube appears to cause a good deal of subsequent cicatricial contraction. Richardson[17] prefers to dispense with all appliances, except a needle and thread, when the patient's condition justifies a deliberate technique. He does not regard Murphy's button as a brilliant success. Hinterstoisser[18] speaks in favour of Murphy's button, and Wiggin[19] eulogises Maunsell's method of intestinal anastomosis, of which he gives full details.

1 Intercol. Med. Journ. of Australasia, June 20, 1896, p. 337. 2 Lancet, June 13, 1896. 3 Amer. Journ. Med. Sci., Sept., 1896; and Boston. Med. and Surg. Journ., Vol. cxxxiv., No. 19. 4 Therap. Gazette, June 15, 1896. 5 Lancet, July 11, 1896, p. 113. 6 Ibidem, July 25, 1896. 7 Med. Reprints, Sept. 15, 1896, p. 115. 8 Lancet, June 13, 1896, p. 1,642. 9 Glasgow Med. Journ., July, 1896, p. 37. 10 Annals of Surg., Aug. 1896. 11 Lancet, July 11, 1896, p. 113. 12 New York Med. Record, June 6, 1896. 13 Med. Week, May 22, 1896. 14 Brit. Med. Journ., June 6, 1696. 15 The Lancet, July 18. 1896. 16 Clinical Journ., Sept. 23, 1896, p. 338. 17 Ibidem, Sept. 19, 1896. 18 Brit. Med. Journ., June 20, 1896; and Wein. Klin. Woch, May 21, 1896. 19 Med. Press and Circular, June 23, 1896, and July 1, 1896.

